# Objective and subjective neurocognitive functioning in functional motor symptoms and functional seizures: preliminary findings

**DOI:** 10.1080/13803395.2023.2245110

**Published:** 2023-09-19

**Authors:** Susannah Pick, L.S. Merritt Millman, Yiqing Sun, Eleanor Short, Biba Stanton, Joel S. Winston, Mitul A. Mehta, Timothy R. Nicholson, Antje A.T.S. Reinders, Anthony S. David, Mark J. Edwards, Laura H. Goldstein, Matthew Hotopf, Trudie Chalder

**Affiliations:** a Institute of Psychiatry, Psychology, and Neuroscience, King’s College London, UK; b Institute of Mental Health, University College London, UK; c South London and Maudsley NHS Foundation Trust, UK

**Keywords:** Cognitive, functional neurological disorder, conversion disorder, psychogenic non-epileptic seizures, functional movement disorder, attention, memory, metacognition, neuropsychological, executive

## Abstract

**Introduction:**

This study aimed to provide a preliminary assessment of objective and subjective neurocognitive functioning in individuals with functional motor symptoms (FMS) and/or functional seizures (FS). We tested the hypotheses that the FMS/FS group would display poorer objective attentional and executive functioning, altered social cognition, and reduced metacognitive accuracy.

**Method:**

Individuals with FMS/FS (n = 16) and healthy controls (HCs, n = 17) completed an abbreviated CANTAB battery, and measures of intellectual functioning, subjective cognitive complaints, performance validity, and comorbid symptoms. Subjective performance ratings were obtained to assess local metacognitive accuracy.

**Results:**

The groups were comparable in age (p = 0.45), sex (p = 0.62), IQ (p = 0.57), and performance validity (p-values = 0.10–0.91). We observed no impairment on any CANTAB test in this FMS/FS sample compared to HCs, although the FMS/FS group displayed shorter reaction times on the Emotional Bias task (anger) (p = 0.01, np2 = 0.20). The groups did not differ in subjective performance ratings (p-values 0.15). Whilst CANTAB attentional set-shifting performance (total trials/errors) correlated with subjective performance ratings in HCs (p-values<0.005, r_s_ = −0.85), these correlations were non-significant in the FMS/FS sample (p-values = 0.10–0.13, r_s_-values = −0.46–0.50). The FMS/FS group reported more daily cognitive complaints than HCs (p = 0.006, g = 0.92), which were associated with subjective performance ratings on CANTAB sustained attention (p = 0.001, r_s_ = −0.74) and working memory tests (p < 0.001, r_s_ = −0.75), and with depression (p = 0.003, r_s_ = 0.70), and somatoform (p = 0.003, r_s_ = 0.70) and psychological dissociation (p-values<0.005, r_s_-values = 0.67–0.85).

**Conclusions:**

These results suggest a discordance between objective and subjective neurocognitive functioning in this FMS/FS sample, reflecting intact test performance alongside poorer subjective cognitive functioning. Further investigation of neurocognitive functioning in FND subgroups is necessary.

## Introduction

Functional neurological disorder (FND) refers to distressing and/or disabling neurological symptoms that have unique features distinct from other neurological and medical disorders, and that are unexplained by neuropathology (APA, 2013). In DSM-5 (APA, 2013), FND includes altered voluntary motor and sensory functions, such as seizures, weakness/paralysis, movement disorders and sensory alterations. However, subjective cognitive symptoms (e.g., memory and attentional difficulties) are also common in individuals diagnosed with functional motor symptoms (FMS) and seizures (FS), and are associated with reduced quality of life and elevated psychological symptoms in these groups (Forejtová et al., 2022; Goldstein et al., 2021; Myers et al., 2014; Věchetová et al., 2018).

An isolated phenotype of subjective cognitive complaints is increasingly recognized, which can be diagnosed as functional cognitive disorder (FCD; Hallett et al., 2022; Stone et al., 2015; Teodoro et al., 2018). In FCD, these subjective cognitive complaints are inconsistent and unexplained by identifiable neuropathology (Ball et al., 2020). It is unclear whether there is overlapping pathophysiology underlying cognitive symptoms in individuals with FMS/FS and those with FCD; however, here we focused specifically on examining neurocognitive functioning in individuals with FMS/FS as the primary diagnosis, given the high rates and potential impact of cognitive complaints in these subgroups.

Explanatory models of FND, including FMS and FS, have emphasized disturbances in neurocognitive processes and associated neurocircuitry, such as executive control (Baslet, 2011; Brown & Reuber, 2016; Perez et al., 2012; Voon et al., 2013), attentional processing (Baslet, 2011; Brown & Reuber, 2016; Edwards et al., 2012), affective information processing and social cognition (Jungilligens et al., 2022; Kozlowska et al., 2015; Pick et al., 2019). Dysfunction in fronto-parietal attention, cognitive/motor control and limbic/salience networks have been emphasized in pathophysiological models of FND (e.g., Drane et al., 2021; Hallett et al., 2022; Pick et al., 2019).

Whilst subjective cognitive complaints are present in many people with FND, empirical evidence for objective impairment in neurocognitive functioning is variable. Numerous studies demonstrated deficits on neurocognitive tasks in FND samples compared to healthy and/or clinical controls. Existing findings include differences on tests of primary attention and/or attentional control in samples with FS (O’Brien et al., 2015; Simani et al., 2020; Strutt et al., 2011), FMS (Roelofs et al., 2003; Věchetová et al., 2022) and mixed FND symptoms (Keynejad et al., 2020; De Vroege et al., 2021). Diminished performance in aspects of executive functioning has been reported in FS (Black et al., 2010; Hamouda et al., 2021; Jungilligens et al., 2020; O’Brien et al., 2015; Simani et al., 2020; Strutt et al., 2011), FMS (Brown et al., 2014; Věchetová et al., 2022; Voon et al., 2013), FCD (Ball et al., 2021) and mixed FND samples (Hammond-Tooke et al., 2018; Kozlowska et al., 2015; De Vroege et al., 2021). There is also emerging evidence of altered social-emotional cognition, particularly in FS samples (Jungilligens et al., 2020; Pick et al., 2016a, 2018a,b; Schönenberg et al., 2015). Finally, learning and memory impairments have also been observed in FS (Hamouda et al., 2021; O’Brien et al., 2015; Strutt et al., 2011), FMS (Brown et al., 2014; Heintz et al., 2013), FCD (Ball et al., 2021) and mixed FND symptoms (Demır et al., 2013; Kozlowska et al., 2015).

In contrast, several studies reported similar or superior performance compared to controls in relevant neurocognitive domains in FMS (Heintz et al., 2013; Voon et al., 2013), FS (Hamouda et al., 2021; Pick et al., 2016a, 2018a,b; Strutt et al., 2011; Tyson et al., 2018) and FCD (Bhome et al., 2019; McWhirter, Ritchie et al., 2022; Teodoro et al., 2023).

Discrepancies between subjective and objective measures of neurocognitive performance have also been reported, indicating that individuals with FND might underestimate their own abilities and performance, both in FS (Breier et al., 1998; Fargo et al., 2004; Prigatano & Kirlin, 2009) and FCD (Bhome et al., 2022; McWhirter, Ritchie et al., 2022; Pennington, Hayre, Newson, & Coulthard, 2015; Teodoro et al., 2023). However, empirical evidence on local and global metacognition in FND is preliminary and there have been mixed findings in FMS (Bègue et al., 2018; Matthews et al., 2020: Verrel et al., 2023) and FCD (Pennington et al., 2021; Teodoro et al., 2023), with a lack of focused metacognitive studies in FS. In addition, frequent cognitive complaints and poor concordance between objective and subjective neurocognitive functioning have also been observed in healthy adults (e.g., Burmester et al., 2016; Crumley et al., 2014; McWhirter, King et al., 2022).

Inconsistencies in the literature in FND may also be explained in part by variability in methodology. Different tests have been employed to assess neurocognitive domains across studies, with some investigators adopting standardized tests with established psychometric properties, and others using unstandardized variants of computerized tasks lacking published psychometric properties. FND samples were compared to normative data or healthy participants in some instances, whereas others reported comparisons with psychiatric and/or neurological controls.

Various confounding variables might also influence neurocognitive testing outcomes in FND, including education, age, medication use, history of head injury, minor structural brain abnormalities, psychiatric (e.g., anxiety, depression) and/or physical (e.g., pain, fatigue, sleep disturbances) comorbidities, negative response biases and reduced engagement/effort.

In summary, individuals with FMS and FS frequently report subjective cognitive symptoms and several objective neurocognitive differences have been implicated in FND more generally, including altered attention, executive functioning, and social cognition. Inconsistencies in previous findings warrant additional research using objective neurocognitive tests alongside measures of subjective neurocognitive functioning, to better understand the nature and relevance of altered neurocognitive functioning in specific FND subgroups.

### Aims

This preliminary study was part of a broader project which combined multimodal research methods to investigate etiological factors and mechanisms in two common FND phenotypes (FMS and FS). Here, we aimed to provide an initial assessment of aspects of objective and subjective neurocognitive functioning in these subgroups, as well as assessing the feasibility and utility of our procedures to inform the design of a subsequent larger-scale project.

An established battery (Cambridge Cognition, 2019) was used to assess aspects of objective neurocognitive performance. We examined subjective neurocognitive functioning (local metacognition) by acquiring participants’ self-evaluative performance ratings for each test. We hypothesized that the FMS/FS group would exhibit poorer objective performance than healthy controls (HCs) in attention and executive functioning, as well as altered social cognition, including reduced facial expression recognition accuracy and enhanced attentional bias toward emotional faces (Pick et al., 2016a, 2018b). We also predicted that the FMS/FS group would display poorer local (i.e., test-specific) metacognitive accuracy. We included a self-report measure of subjective daily cognitive complaints to test the hypothesis that the FMS/FS group would report poorer global subjective cognitive functioning.

We aimed to control for the potential confounding influence of age, sex, education, medication and general intellectual abilities. A performance validity test was administered to assess task engagement. A final aim was to explore relationships between aspects of neurocognitive functioning and clinical features in the FMS/FS sample.

## Materials and methods

### Participants

This cross-sectional between-group study included 16 individuals diagnosed with FMS/FS and 17 healthy controls. The sample size was determined by our aims of piloting the procedures and estimating effect sizes on the measures.

Recruitment of participants with FMS/FS took place online via advertisement circulated by patient support organizations (e.g., FND Hope UK, FND Action) and social media platforms. Advertisements for control participants were circulated on local community webpages. Control participants were selected to match the groups on relevant sociodemographic characteristics, including age, gender, and years of education.

Inclusion criteria for all participants were: normal or corrected eyesight, aged 18–65 years, and fluency in English. Participants in the FMS/FS group were required to provide medical documentation demonstrating evidence of a primary diagnosis of FMS and/or FS, according to DSM-5 criteria. Documentation was verified by the principal investigator (SP), who also assessed whether participants met DSM-5 criteria for FND at entry to the study during an in-depth baseline interview (see below). Ambiguous medical documentation was reviewed by a consultant neurologist (BS). To ensure that our sample was representative of the broader FND population, individuals reporting additional functional neurological symptoms were not excluded from the sample, but only those with FMS or FS as their primary diagnosis were eligible for inclusion.

Exclusion criteria for all participants were: a diagnosis of major cardiovascular (e.g., heart disease), psychiatric (e.g., psychosis, alcohol or substance dependence) or neurological disorder (e.g., epilepsy, multiple sclerosis), and physical symptoms or disability impairing ability to perform the tasks. Potential participants taking medications that might significantly impair attention and concentration were also excluded (e.g., daily/multiple opiate analgesics). A current or historical diagnosis of functional neurological disorder was an additional exclusion criterion in the HC group.

This study conformed to the World Medical Association Declaration of Helsinki and was approved by the King’s College London Health Faculties High-Risk Research Ethics Sub-Committee in June 2022 (ref: HR/DP-21/22-28,714).

### Materials & measures

#### Wechsler Abbreviated Scale of Intelligence – 2nd edition (WASI-II; Wechsler, 2011)

The two sub-test version of the WASI-II (Matrix Reasoning, Vocabulary) assessed non-verbal and verbal intellectual abilities respectively. The two sub-test version yields a full-scale intelligence quotient (IQ) score (FSIQ-2) which has excellent internal consistency (0.94), test-retest stability (.94) and inter-rater reliability (.95-.99; Wechsler, 2011).

#### Cambridge Neuropsychological Test Automated Battery (CANTAB) Connect (Cambridge Cognition, 2019)

The original CANTAB automated test battery has sound psychometric properties in psychiatric, neurological and healthy samples (e.g., Fray et al., 1996; Robbins, 1994; Robbins et al., 1998). The CANTAB Connect application allowed the tests to be administered using a touchscreen device (iPad). Table 1 presents the CANTAB tests included; additional details of outcome measures are provided in Supplementary Table 1. To minimize the testing burden for participants, we selected only those tests measuring neurocognitive functions of most potential relevance to the etiology and mechanisms of FMS and FS, as well as tests assessing basic psychomotor/information processing speed.

**Table 1.** Description of CANTAB connect tests.

Test	Cognitive Domain	Description	Outcome variables
Motor Screening	Sensorimotor speed	2-minute test. Participants detect and manually select colored crosses appearing in varied spatial locations onscreen, as quickly as possible.	Motor Mean Latency Total correct/incorrect
Reaction Time	Cognitive and motor response speed	3-minute test. Participants are asked to hold their finger on a central circle at the bottom of the screen until one of five circles at the top of the screen lights up. Participants must release the lower circle and manually select the target upper circle as quickly as possible.	Reaction Time Movement Time Total Error Score
Rapid Visual Information Processing	Attention (sustained)	7-minute test (approx.). Participants required to detect target digit sequences (e.g., 5-3-7) appearing within a stream of individually presented digits (100/minute). Participants must indicate the occurrence of the target sequence by manually selecting a button onscreen as quickly as possible.	Response Latency Ability score Total misses Probability of Hit False Alarm
Spatial Span	Working memory (visuospatial)	5-minute test. Participants are presented with sequences of squares changing color one-by-one in a variable spatial pattern. Participants are required to repeat each sequence manually, either in the same order, or backwards. Task difficulty increases as the task progresses (2–9 squares in a sequence).	Forward/reverse span length Forward/reverse errors
Intra-Extra Dimensional Set Shift	Attentional set-shifting, cognitive flexibility, visual discrimination	7-minute test. Adaptation of the Wisconsin Card Sorting Test. Pink shapes and white lines are presented, according to an implicit rule that the participant must identify. Participants are asked to select the box that they think adheres to the current rule and they are given feedback on each trial (“Correct” or “Incorrect”). Once the participant has correctly responded six times, the rule changes and the participant must identify a new rule. The rules either change within a dimension (i.e., intra-dimensional set shift), or the rule changes to focus on a different dimension (i.e., extra-dimensional set shift). Task difficulty increases throughout.	Total/adjusted errors Total trials completed Completed Stage Trials/Errors Stages completed Response latency
Stop Signal Task	Response inhibition	14-minute test. Arrows presented individually, pointing to the left or right. Participants must indicate the direction of the arrow as quickly as possible by pressing a left or right button. When an auditory stimulus is also present, participants must withhold the button press (response inhibition). The task is adaptive, with variable stop-signal delay dependent on the participant’s performance.	Errors (Go/Stop trials) Missed trials Stop signal reaction time
Emotional Bias Task	Social cognition	4-minute test. Each trial involves 150-millisecond presentation of a morphed emotional face, which vary in intensity from one emotion to another. Two versions of the task were included (happy-angry, happy disgust). Participants are given a forced-choice option to select which emotion they perceived.	Bias point: proportion of assessed trials where the subject selected “Happy,” (adjusted to 0–15) Reaction time by emotion
Emotion Recognition Test	Social cognition	6–10 minute test. Assesses recognition of six emotional facial expressions (anger, sadness, fear, disgust, surprise, happiness). Each trial involves a 200-millisecond presentation of an emotional face. Participants are asked to select one of six emotion labels to report which emotion they perceived, as quickly as possible.	Total hits/Unbiased hits Reaction times False alarms

#### Subjective performance ratings

Participants were asked to rate their performance on each neurocognitive test immediately on completion using a 7-point Likert scale, where 1 = Very poor performance; 2 = Poor performance; 3 = Below average; 4 = Average; 5 = Above average; 6 = Superior; 7 = Very superior. This allowed assessment of the degree to which participants could accurately appraise their performance on each task (i.e., task-specific metacognitive accuracy).

#### Performance validity

The Medical Symptom Validity Test (MSVT; Green, 2003) was administered. Validity outcomes were percentage correct scores for immediate and delayed recall, and immediate-delayed consistency scores. Delayed paired associates and free recall scores were memory indices. The cutoff score for test failure is ≤85% on any validity outcome. The MSVT has satisfactory psychometric properties (e.g., Green & Flaro, 2016; Green et al., 2011; Howe & Loring, 2008).

#### Cognitive failures questionnaire (*Broadbent* et al., 1982)

This self-report measure assessed the frequency of subjective cognitive complaints in daily life. The 25 items assess the frequency of common cognitive errors over the preceding six months. Higher scores indicate poorer subjective cognitive functioning. The Cognitive Failures Questionnaire (CFQ) displays adequate psychometric properties, including good internal consistency (0.79–0.89; Bridger et al., 2013; Broadbent et al., 1982; De Paula et al., 2017; Wallace et al., 2002).

#### Clinical self-report measures

We administered a range of self-report measures (Supplementary Table 2) to assess the following:

- Presence/absence of subjective FND symptoms, plus severity and impact ratings (bespoke questionnaire designed for the study, see Supplementary Table 3)
- Common physical symptoms (Patient Health Questionnaire-15; Kroenke & Spitzer, 2002)
- Depression (Patient Health Questionnaire-9; Kroenke et al., 2001)
- Anxiety (Generalized Anxiety Disorder-7; Spitzer et al., 2006)
- Dissociation (Multiscale Dissociation Inventory; Briere, 2002), (Somatoform Dissociation Questionnaire-20; Nijenhuis et al., 1996)
- Alexithymia (Toronto Alexithymia Scale-20; Bagby et al., 1994)
- Autistic spectrum traits (Autistic Quotient; Baron-Cohen et al., 2001)
- Traumatic experiences (Traumatic Experiences Checklist; Nijenhuis et al., 2002)
- Illness-related cognitions (Brief Illness Perception Questionnaire; E. Broadbent et al., 2006)
- General functioning (Work & Social Adjustment Scale; Mundt et al., 2002)
- Health-related quality-of-life (HRQoL; 36-item Short Form survey; Hays et al., 1993)

### Procedure

Data collection occurred between July and October 2022. All data were collected by an academic/experimental psychologist (SP) with extensive experience of neurocognitive testing in FND samples.

Participants who appeared eligible at first contact with the research team provided written informed consent before undergoing a detailed screening interview, which elicited information on participants’ sociodemographic characteristics, medical history and eligibility. An abbreviated structured clinical interview (SCID-5-RV; First et al., 2016) screened for diagnoses relevant to the exclusion criteria.

Eligible participants completed clinical self-report questionnaires online using Qualtrics software (https://www.qualtrics.com/uk/) within 48 hours prior to attending a testing session at King’s College London. All neurocognitive testing took place in a purpose-built laboratory between 10am-12pm. Participants were compensated with a £50 shopping voucher at the end of the session, which included several additional experimental tasks reported elsewhere.

## Data analyses

The data were analyzed using *R* (Version 4.1.0, 2021) and/or SPSS (IBM, 2021), verified independently by two members of the research team (SP/LSMM).

Shapiro-Wilk tests and QQ-plots were used to evaluate the assumption of normality. Outlying scores of 2.5 standard deviations above/below the group mean for a given test were excluded from analysis if their inclusion significantly altered the test outcome. Excluded outliers and missing data are detailed in the relevant Results tables.

Between-group comparisons for categorical variables were analyzed with Fisher’s exact or chi-squared tests. Independent samples t-tests were used for between-group comparisons with normally distributed continuous variables. Levene’s test assessed equality of variances. Wilcoxon rank sum tests were conducted for continuous variables that were not normally distributed. One-tailed tests were conducted to test directional hypotheses, with alpha set at p ≤ 0.05. As such, effects observed in the inverse direction to the hypotheses are not interpreted/discussed (Howell, 1997).

Mixed analyses of variance (ANOVAs) were used for the CANTAB Emotional Bias Tasks (EBTs) and the Emotion Recognition Test (ERT) because these tests included both within- and between-group factors. The sphericity assumption was checked with Mauchly’s test and Greenhouse-Geiser corrections applied where violations were detected. Mixed ANOVAs used for the EBTs had one between-group factor (diagnosis: FMS/FS vs HC) and one within-group factor (emotion: anger/disgust vs happiness). The mixed ANOVA used for the ERT had one between-group factor with two levels (diagnosis: FMS/FS vs HC) and one six-level within-group factor (emotion: anger, disgust, happiness, sadness, fear, surprise). Where significant main effects or interactions were observed, post-hoc t-tests were conducted with Bonferroni corrections.

Exploratory correlations were conducted with Pearson’s r (normally distributed variables) or Spearman’s rho coefficients (non-normally distributed variables), to examine potential relationships between neurocognitive outcome variables and self-reported cognitive functioning, performance ratings, and clinical features. A more stringent alpha value (p ≤ 0.005) was adopted to evaluate significance in these analyses, to control for probable elevation in familywise error due to multiple testing. This pragmatic approach was applied to reduce the likelihood of Type 1 errors resulting from the large number of variables evaluated. We did not use a formal correction method in these analyses because Type 2 error rates can be inflated when more conservative methods are used with large numbers of exploratory tests (e.g., Bonferroni). Only tests with p ≤ 0.005 are presented in the relevant Results sections.

Effect sizes were calculated with Hedges’ g (Hedges & Olkin, 1985) for t-tests due to the small sample size, *r* values for Wilcoxon, Pearson’s and Spearman’s tests, and partial-eta squared for mixed ANOVAs.

## Results

### Sociodemographic and clinical characteristics

The FMS/FS and HC groups did not differ significantly on most sociodemographic features; however, a smaller proportion of participants in the FMS/FS group were in employment or education, and a greater proportion reported taking medication and (comorbid) physical and mental health diagnoses, compared to HCs (Table 2 and Supplementary Table 4). All participants in the FND group reported at least one other FND symptom in addition to their primary FMS/FS diagnosis, most commonly sensory or cognitive symptoms. The average FND symptom severity and impact ratings were in the moderate range.

**Table 2.** Sociodemographic and clinical characteristics.

	FND (n = 16)	HC (n = 17)	Comparison statistics
Self-reported FND symptoms: n (%)	Motor = 16 (100) Seizures = 7 (44) Sensory = 16 (100) Speech/swallowing = 9 (56) Dizziness = 14 (81) Cognitive = 14 (88) Other = 8 (50) Multiple = 16 (100%)		
FND average symptom severity/impact (1–7): M (SD)	Severity = 4.17 (0.93) Impact = 4.19 (0.79)		
Age (years): M (SD)	36.1 (10.8)	39.0 (11.0)	t(31) = 0.76, p = 0.45, g = 0.26
Sex: n (%)	F = 12 (75) M = 4 (25)	F = 13 (76) M = 4 (24)	p = 0.62 (Fisher’s exact)
Handedness: n (%)	R = 14 (88)	R = 15 (88)	p = 1.0 (Fisher’s exact)
Relationship status – married/cohabiting: n (%)	10 (63)	8 (47)	p = 0.49 (Fisher’s exact)
Ethnicity: n (%)	White: 13 (81) Black: 0 (0) Asian: 0 (0) Other: 3 (19)	White: 12 (71) Black: 3 (18) Asian: 2 (12) Other: 0 (0)	p = 0.69 (Fisher’s exact: White/nonwhite)
Education – post-compulsory: n (%)	15 (94)	17 (100)	p = 0.49 (Fisher’s exact)
Occupational status – employed/student: n (%)	6 (38)	16 (94)	p 0.001 (Fisher’s exact)
Current physical health diagnosis: n (%)	11 (69)	4 (24)	p = 0.01 (Fisher’s exact)
Current mental health diagnosis: n (%)	10 (63)	1 (6)	p < 0.001 (Fisher’s exact)
Current medication use: n (%)	15 (94)	5 (29)	p < 0.001 (Fisher’s exact)

Key: M = mean; SD = standard deviation

### General intellectual functioning

Full-scale intelligence quotient (WASI-II FSIQ-2) scores were comparable in participants with FND and HCs, with the mean scores falling in the average range for both groups (Table 3).

**Table 3.** General intellectual functioning and performance validity scores.

	FND (n=16)	HC (n=17)	Comparison statistics
WASI-II FSIQ-2 scores: M (SD)	104.6 (10.7)	106.6 (9.2)	t(31)=0.58, p=0.57, g=0.20
*Vocabulary T scores: M (SD)*	54.1 (5.7)	55.0 (8.6)	t(31)=0.34, p=0.73, g=0.12
*Matrix Reasoning T scores: M (SD)*	51.4 (8.6)	52.8 (4.9)	t(24)=0.56, p=0.58, g=0.19
Medical Symptom Validity Test			
*Immediate Recall % Correct: Mdn (IQR)*	100.0 (0.0)	100.0 (0.0)	W=136.0, p=1.00, r=0.00
*Delayed Recall % Correct:* *Mdn (IQR)*	100.0 (0.0)	100.0 (0.0)	W=120.0, p=0.18, r=0.24
*Consistency %: Mdn (IQR)*	100.0 (0.0)	100.0 (0.0)	W=120.0, p=0.18, r=0.24
*Paired Associates % Correct: Mdn (IQR)*	100.0 (0.0)	100.0 (0.0)	W=120.5, p=0.34, r=0.17
*Free Recall % correct:* *M (SD)*	83.1 (12.8)	82.6 (11.1)	t(31)=-.12, p=0.91, g=0.04
*MSVT Pass: n (%)*	16 (100)	17 (100)	-

Key: IQR = interquartile range; M = mean; Mdn = median; SD = standard deviation; WASI-II FSIQ-2 = Wechsler Abbreviated Scale of Intelligence–Full-Scale Intelligence Quotient 2 sub-test

### Performance validity testing

There were no significant group differences on any Medical Symptom Validity Test (MSVT) subscale (Table 3). All participants achieved scores above the cutoff on the validity and memory subscales.

### CANTAB test performance

Table 4 displays all CANTAB test statistics.

**Table 4.** CANTAB Connect test statistics.

	FND (n = 16)	HC (n = 17)	Comparison statistics
Motor Screening Test *Mean Motor Latency*: M (SD)	862.2 (189.7)	751.8 (199.7)	t(31)=-1.63, p=0.11, g=0.55
Reaction Time Test			
*Mean Reaction Time (ms): M (SD)*	382.2 (41.5)	361.1 (32.7)	t(31)=-1.62, p=0.12, g=0.55
*Mean Movement Time (ms): Mdn (IQR)*	252.2 (54.6)	217.9 (55.0)	W=92.0, p=0.12, r=0.27
Rapid Visual Information Processing			
*Mdn Response Latency (ms): Mdn (IQR)*	403.5 (40.1)	440.0 (85.4) (n=16)*^1^	W=179.5, p=0.05, r=0.3
*RVIP Ability: M (SD)*	0.89 (0.05)	0.91 (0.05) (n=16)*^1^	t(30)=0.81, p=0.43, g=0.28
*Total Misses: M (SD)*	22.4 (10.3)	21.0 (12.2)	t(31)=-0.37, p=0.72, g=0.12
*Probability of Hit: M (SD)*	0.58 (0.19)	0.61 (0.23)	t(31)=0.37, p=0.72, g=0.12
*Probability of False Alarm: Mdn (IQR)*	0.006 (0.008)	0.004 (0.003) (n=16)*^1^	W=96.0, p=0.23, r=0.21
Spatial Span			
*Forward Span Length: Mdn (IQR)*	7.0 (1.0)	7.0 (3.0)	W=156.5, p=0.46, r=0.13
*Forward Errors: M (SD)*	15.9 (5.1)	17.2 (8.8)	t(26.0)=0.55, p=0.59, g=0.18
*Reverse Span Length: Mdn (IQR)*	6.0 (1.5) (n=15)*^1^	6.0 (1.0)	W=107.0, p=0.44, r=0.13
*Reverse Errors: M (SD)*	14.4 (3.5) (n=15)*^1^	12.2 (5.7)	t(27.0)=-1.34, p=0.19, g=0.45
Intra-Extra Dimensional Set Shift			
*Total Errors: Mdn (IQR)*	14.0 (7.8) (n=12)*^4^	12.0 (8.5) (n=10)*^7^	W=55.0, p=0.77, r=0.05
*Adjusted Errors: Mdn (IQR)*	16.0 (28.5)	22.0 (47.0)	W=152.0, p=0.58, r=0.10
*Total Trials Completed: M (SD)*	80.7 (16.6) (n=12)*^4^	75.4 (8.3) (n=10)*^7^	t(20)=-0.26, p=0.80, g=0.11
*Completed Stage Trials: M (SD)*	70.0 (25.0)	71.2 (15.7)	t(31)=0.17, p=0.87, g=0.06
*Completed Stage Errors: Mdn (IQR)*	12.0 (7.75)	12.0 (11.0)	W=146.5, p=0.72, r=0.06
*Stages Completed: Mdn (IQR)*	9.0 (0.5)	9.0 (2.0)	W=112.5, p=0.33, r=0.17
*Response Latency (ms): Mdn (IQR)*	112341 (42752) (n=12)*^4^	125164 (44040) (n=10)*^7^	W=73.0, p=0.42, r=0.14
Stop Signal Task			
*Errors Go Trials: Mdn (IQR)*	0.0 (1.0)	0.0 (2.0)	W=138.0, p=0.95, r=0.01
*Errors Stop Trials: Mdn (IQR)*	38.5 (8.5)	39.0 (6.0)	W=154.5, p=0.52, r=0.11
*Number of Missed Trials: Mdn (IQR)*	4.0 (5.3)	2.0 (5.0)	W = 105.5, p=.28, r=.19
*Stop Signal Reaction Time (ms): M (SD)*	241 (41.2)	249 (54.2)	t(31)=.44, p=.66, g=.16
Emotional Bias Task - Anger			
*Bias Point: M (SD)*	8.56 (1.4)	8.82 (1.1)	t(31)=0.60, p=0.55, g=0.21
*Mdn reaction time x Emotion: M (SD)*			*ANOVA* *Emotion*: F(1, 30)=4.57, p=0.04, np2=0.13 *Group:* F(1, 30)=7.59, p=0.01, np2=0.20 *Group x Emotion:* F(1, 30)=0.24, p=0.63, np2=0.008 (FND n=15^#1^, HC n=17)
*Anger (ms)*	772.4 (161.1)	958.4 (255.1)	
*Happiness (ms)*	726.3 (122.7) (n=15)^#1^	885.1 (198.6) (n=17)	
Emotional Bias Task - Disgust			
*Bias Point: M (SD)*	8.16 (.86) (n=15)*	7.94 (1.16)	t(30)=-0.59, p=0.56, g=0.20
*Mdn reaction time x Emotion: M (SD)*			*ANOVA* *Emotion:* F(1, 28)=1.4, p=0.24, np2=0.05 *Group:* F(1, 28)=3.1, p=0.09, np2=0.10 *Group x Emotion:* F(1, 28)=1.42, p=0.24, np2=0.05 (FND n=14*^1#1^, HC n=16^#1^)
*Disgust (ms)*	684.3 (95.1)	789.3 (170.0)	
*Happiness (ms)*	723.4 (97.6) (n=14)*^1#1^	789.4 (168.5) (n=16)^#1^	
Emotion Recognition Test			
*Total Hits: Mdn (IQR)*	59.5 (8.25)	59.5 (7.0) (n=16)*^1^	W=145.5, p=0.52, r=0.11
*Total Hits x Emotion: M (SD)*			*ANOVA* *Emotion*: F(5, 145)=24.3, p<.001, np2=0.46 *Group:* F(1, 29)=0.05, p=0.83, np2=0.002 *Group x Emotion:* F(5, 145)=2.31, p=0.047, np2=0.07 (FND n=15^#1^, HC n=16*^1^)
*Anger*	8.1 (2.2)	7.3 (2.4)	
*Disgust*	9.8 (3.2)	10.8 (3.3)	
*Fear*	7.2 (2.4)	7.4 (3.2)	
*Happiness*	12.0 (2.2)	12.4 (1.6)	
*Sadness*	11.4 (2.8)	9.6 (4.1)	
*Surprise*	11.1 (1.8)	11.9 (1.7) (n=16)*^1^	
*Unbiased Hit Rate x Emotion: M (SD)*			*ANOVA* *Emotion:* F(5, 150)=22.5, p<0.001, np2=0.43 *Group:* F(1, 30)=0.02, p=0.88, np2=0.001 *Group x Emotion:* F(5, 150)=2.22, p=0.06, np2=0.07 (FND n=16; HC n=16*^1^)
*Anger*	0.42 (0.17)	0.41 (0.17)	
*Disgust*	0.42 (0.19)	0.50 (0.22)	
*Fear*	0.32 (0.15)	0.31 (0.22)	
*Happiness*	0.59 (0.13)	0.66 (0.11)	
*Sadness*	0.54 (0.14)	0.45 (0.20)	
*Surprise*	0.5 (0.09)	0.50 (0.12) (n=16)*^1^	
*False Alarms x Emotion: M (SD)*			*ANOVA* *Emotion:* F(3.3, 97.5^$^)=4.87, p=0.003, np2=0.14 *Group:* F(1, 30)=0.006, p=0.94, np2=0.00 *Group x Emotion:* F(3.3, 97.5^$^)=1.54, p=0.21, np2=0.05 (FND n=16; HC n=16*^1^)
*Anger*	2.8 (2.5)	2.1 (3.0)	
*Disgust*	7.4 (6.3)	6.3 (4.8)	
*Fear*	4.5 (3.8)	6.3 (4.6)	
*Happiness*	5.3 (5.2)	3.4 (2.8)	
*Sadness*	5.1 (3.5)	4.4 (3.4)	
*Surprise*	5.4 (2.6)	8.3 (5.5) (n=16)*^1^	
*Mdn reaction time – Correct responses (ms): Mdn (IQR)*	1039.0 (227.0)	1048.5 (188.0) (n=16)*^1^	W=141.5, p=0.62, r=0.08
*Mdn reaction time (correct) x Emotion (ms): M (SD)*			*ANOVA* *Emotion:* F(2.3, 67.9^$^)=7.88, p<0.001, np2=0.21 *Group:* F(1, 29)=0.13, p=0.72, np2=0.01 *Group x Emotion:* F(2.3, 67.9^$^)=1.02, p=0.38, np2=0.03 (FND n=15*^1^, HC n=16*^1^)
*Anger*	1152.5 (521.0)	1201.6 (405.2)	
*Disgust*	1301.2 (470.1)	1175.9 (223.9)	
*Fear*	1362.7 (362.9)	1660.9 (1155.2)	
*Happiness*	926.0 (289.3)	873.4 (172.1)	
*Sadness*	1142.8 (237.8)	1082.6 (237.7)	
*Surprise*	948.1 (272.1) (n=15) *^1^	1050.5 (463.1) (n=16)*^1^	

Key: ANOVA=Analysis of Variance; IQR = interquartile range; M = mean; Mdn = median; ms = milliseconds; SD = standard deviation;

*sample size diverges from total n due to missing data

#sample size diverges from total n due to outlier exclusion

^$^Greenhouse-Geiser correction for non-sphericity

#### Sensorimotor and information processing speed

There were no significant between-group differences on any outcome on the Motor Screening and Reaction Time (RT) tests.

#### Attention, working memory and executive functioning

Compared to HCs, the FMS/FS group displayed no significant impairments in performance on the Rapid Visual Information Processing (RVIP, sustained attention), Spatial Span (working memory), Intra-Extra Dimensional Set-Shift (cognitive flexibility/set-shifting) and Stop Signal (response inhibition) tasks.

#### Social cognition

There were no significant between-group differences on most outcomes of the Emotional Bias Tasks (EBT), including Bias Point scores. However, on the EBT-Anger version, the mixed ANOVA yielded significant main effects of emotion and group on RTs (both large effect sizes). The group effect reflected shorter RTs in the FMS/FS group (estimated marginal mean = 749.4 ms, standard error = 45.6 ms) relative to HCs (estimated marginal mean = 921.8 ms, standard error = 42.9 ms). The main effect of emotion was due to shorter RTs for happiness (estimated marginal mean = 805.7 ms, standard error = 29.7 ms) compared to anger (estimated marginal mean = 865.4 ms, standard error = 38.3 ms). The group x emotion interaction was not significant.

There was a significant main effect of emotion on hit rates on the Emotion Recognition Test (ERT; large effect size), with the highest hit rates observed for happiness and surprise, and the lowest for anger and fear. Post-hoc t-tests showed that anger and fear had lower accuracy than all other emotions (all p-values ≤0.006). Hit rates for happiness were significantly greater than anger (p < 0.001), disgust (p = 0.03) and fear (p < 0.001), but not sadness (p = 0.25) or surprise (p = 0.71). The group x emotion interaction was also significant (medium effect size) for ERT hit rates; however, post-hoc t-tests did not reveal deficits in recognition of any facial emotion in the FMS/FS group, relative to HCs. When the ERT hit rate analysis was rerun with the CANTAB “Unbiased Hit Rates” outcome variable, the emotion x group interaction was no longer significant (p = 0.06). The main effect of group was not significant for ERT hit rates or unbiased hit rates.

There was a significant main effect of emotion on false alarms in the ERT (large effect size), reflecting significantly lower rates of false alarms for anger compared to disgust (p = 0.002), fear (p = 0.02), and surprise (p < 0.001). The effect of group and group x emotion interactions were not significant for ERT false alarms.

The main effect of emotion on RTs was significant on the ERT (large effect size). Post-hoc t-tests revealed that RTs were significantly shorter for happiness compared to anger (p = 0.011), disgust (p < 0.001), fear (p = 0.002), and sadness (p = 0.006), but not surprise (p = 1.0). The effect of group and group x emotion interactions were not significant for ERT RTs.

### Subjective neurocognitive functioning

The groups did not differ in their test-specific subjective performance ratings for any neurocognitive test (Table 5). Nevertheless, the FMS/FS group reported significantly more frequent daily cognitive complaints on the Cognitive Failures Questionnaire (CFQ) (M = 55.4, SD = 22.0) relative to HCs (M = 38.1, SD = 14.4), with a large effect size (t(31) = −2.69, p = 0.006, g = 0.92).

**Table 5.** Subjective performance ratings.

	FND (Total n = 16) Mdn (IQR)	HC (Total n = 17) Mdn (IQR)	Comparison statistics
WASI-II	4.5(1.0)	4.5 (0.5)	W=171.0, p=0.19, r=0.23
Motor Screening Test	5.5 (1.25)	6.0 (1.0)	W=143.5, p=0.79, r=0.05
Reaction Time	5.0 (1.0) (n=15)	4.0 (1.0)	W=112.5, p=0.55, r=0.11
Rapid Visual Information Processing	3.5 (1.0)	3.0 (1.0)	W=133.0, p=0.92, r=0.02
Spatial Span			
*Forward*	4.0 (2.0)	4.0 (1.0)	W=128.0, p=0.78, r=0.05
*Reverse*	4.0 (2.0) (n=15)*	4.0 (1.0)	W=130.5, p=0.92, r=0.02
Intra-Extra-Dimensional Set Shift	5.0 (2.0)	4.0 (1.0)	W=97.0, p=0.15, r=0.25
Stop Signal Task	4.0 (0.5) (n=15)*	4.0 (1.0)	W=132.0, p=0.86, r=0.03
Emotion Bias Task			
*Anger*	4.5 (1.0)	5.0 (1.0)	W=155.0, p=0.48, r=0.12
*Disgust*	5.0 (1.0) (n=15)*	5.0 (1.0)	W=141.5, p=0.59, r=0.13
Emotion Recognition Test	4.0 (1.3)	4.0 (1.0) (n=16)*	W=140.0, p=0.65, r=0.08
CANTAB Average	4.3 (0.78)	4.3 (0.68)	W=140.5, p=0.89, r=0.03
Medical Symptom Validity Test	5.0 (1.0)	5.0 (1.0)	W=141.5, p=0.85, r=0.03

Key: CANATB = Cambridge Neuropsychological Test Automated Battery; FND = functional neurological disorder; HC = healthy controls; IQR = interquartile range; Mdn = median; W = Wilcoxon’s W; WASI-II = Wechsler Abbreviated Scale of Intelligence – Second edition

*sample size diverges from total n due to missing data

### Exploratory analyses

#### Subjective performance ratings and objective test performance

In HCs, Intra-Extra-Dimensional Set Shift (IEDSS) subjective performance ratings were strongly correlated with total trials completed (r_s_ = −0.85, p = 0.002) and total errors (r_s_ = −0.85, p = 0.001). However, in the FMS/FS group, correlations were non-significant and only moderate in magnitude for both total trials (r_s_ = −0.46, p = 0.13) and total errors (r_s_ = −0.50, p = 0.10). Nevertheless, the coefficients did not differ between groups for total trials (z = 1.51, p = 0.07) and total errors (z = 1.56, p = 0.06).

#### Subjective test-specific performance ratings and daily cognitive complaints

Daily subjective cognitive complaints (CFQ scores) were negatively associated with subjective performance ratings for the CANTAB RVIP (r_s_ = −0.74, p = 0.001) and Spatial Span Forward (r_s_ = −0.75, p < 0.001) tests in the FMS/FS group, but not in HCs.

#### Daily cognitive complaints and clinical variables

In the FMS/FS group, CFQ scores were positively correlated with somatoform dissociation (r_s_ = 0.70, p = 0.003) and aspects of psychological dissociation, specifically disengagement (r_s_ = 0.85, p < 0.001), derealization (r_s_ = 0.74, p < 0.001), emotional constriction (r_s_ = 0.71, p = 0.002), and memory disturbance (r_s_ = 0.67, p = 0.005). CFQ scores were also strongly associated with depression scores in the FMS/FS sample (r_s_ = 0.70, p = 0.003).

#### Subjective performance ratings and clinical variables

Subjective performance ratings for the MSVT were negatively correlated with PHQ-9 depression (r_s_ = −0.72, p = 0.002) and B-IPQ Emotional Response scores (r_s_ = −0.66, p = 0.005) in the FMS/FS group. Subjective performance ratings on the EBT–Anger version were also negatively associated with B-IPQ Illness Concern scores (r_s_ = −0.78, p < 0.001) in the FMS/FS group. Performance ratings for the Spatial Span (Reverse) task were negatively correlated with FND symptom ratings (r_s_ = 0.-74, p = 0.002).

## Discussion

This study provided a preliminary investigation of aspects of objective and subjective neurocognitive functioning in patients with FMS/FS compared to a healthy control (HC) group. Contrary to our hypotheses, this FMS/FS sample exhibited no impairments compared to HCs on objective tests of attention and executive functioning. Whilst we observed no objective impairment in facial emotion recognition and no overall attentional bias for facial anger or disgust in the FMS/FS group, they displayed reduced RTs on the anger variant of the emotion bias task, suggesting possible attentional hypervigilance on this task.

We observed no absolute group difference in task-specific subjective performance ratings; however, a possible metacognitive difference emerged specifically for attentional set-shifting performance. The FMS/FS sample reported worse subjective cognitive functioning in daily life, which was associated with test-specific subjective performance ratings for sustained attention and working memory tasks. Furthermore, daily cognitive complaints were positively associated with depression and dissociation.

These results suggest a discordance between generally intact performance on objective tests of attention, executive functioning and social cognition, and global subjective cognitive complaints in this FMS/FS sample. The results also indicate possible local metacognitive alterations specifically for attentional set-shifting. Furthermore, global subjective cognitive complaints were linked to psychological symptom burden and domain-specific metacognition in this sample, rather than objective impairments. These findings share similarities with findings in functional cognitive disorder (FCD), in which marked subjective cognitive complaints are not reflected in diminished objective test performance (e.g., Bhome et al., 2019; McWhirter, Ritchie et al., 2022; Pennington, Hayre, Newson, & Coulthard, 2015), suggesting a need for direct comparisons of these subgroups in future studies.

### Objective neurocognitive test performance

The FMS/FS and HC groups were comparable in FSIQ-2 scores and all participants passed the Medical Symptom Validity Test (MSVT), suggesting intact intellectual functioning and adequate task engagement in the FM/FS group, thereby eliminating these as potential confounds.

#### Sensorimotor and information processing speed

Whilst some previous studies reported deficits in information processing speed and/or sensorimotor performance in FMS (Věchetová et al., 2022) and mixed FND samples (De Vroege et al., 2021), we observed no significant differences in this FMS/FS sample. These functions are likely to be influenced by the specific nature and severity of FND symptoms in any given sample, along with possible medication effects. These functions should be accounted for when examining other neurocognitive outcomes in FND samples.

#### Attention and executive functioning

Contrary to our predictions, we observed no objective deficits in attention and executive control in this FMS/FS sample, as measured with several CANTAB tests.

The lack of objective impairments on the Rapid Visual Information Processing (RVIP) test indicated that the FMS/FS group did not experience objective difficulties with *sustained attention*. These findings were unexpected in the context of previous studies reporting impairments in sustained attention in FS (O’Brien et al., 2015; Simani et al., 2020; Strutt et al., 2011), FMS (Roelofs et al., 2003) and mixed FND samples (Kozlowska et al., 2015; De Vroege et al., 2021).

The similar performance in FMS/FS and HC groups on the Intra-Extra Dimensional Set-Shift (IEDSS) test points toward intact *attentional set-shifting* and *cognitive flexibility* in this FMS/FS group. Again, this negates the hypothesized deficit in executive control, but is consistent with another study that used the CANTAB IEDSS task in an FS sample (O’Brien et al., 2015). Similarly, no significant impairments in FMS and/or FS samples have been observed on the Wisconsin Card Sorting Task and the Stroop Color-Word test in several studies (Black et al., 2010; Heintz et al., 2013; Pick et al., 2018a; Voon et al., 2013).

This FMS/FS sample did not display significant difficulties on the Spatial Span tests, pointing toward intact visuospatial *working memory* capacity. Some previous studies reported diminished performance on spatial working memory in FS (O’Brien et al., 2015; Strutt et al., 2011) and digit span tests in individuals with FMS, FS and mixed FND (Demır et al., 2013; Hamouda et al., 2021; Kozlowska et al., 2015; Strutt et al., 2011; De Vroege et al., 2021). However, others found no group differences on working memory span tasks in FS (Özer Çelik et al., 2015; Tyson et al., 2018) and FCD (McWhirter, Ritchie et al., 2022). It would be valuable to assess both types of working memory in future studies in FND samples, with additional tests beyond digit and spatial span tasks.

Consistent with Hammond-Tooke et al. (2018), we did not detect any marked impairments in *response inhibition* in this FMS/FS sample, assessed with the Stop Signal Task (SST). These findings conflict with previous reports of differences in response inhibition in some FS, FMS and mixed FND samples, measured with Go/No-Go tests (Hammond-Tooke et al., 2018; Jungilligens et al., 2020; Kozlowska et al., 2015; Voon et al., 2013). The SST assesses “action cancellation,” whereas Go/No-Go tests assess “action restraint” (Hammond-Tooke et al., 2018; Voon et al., 2013); therefore, these findings suggest that FND may be associated specifically with difficulties at the stage of action restraint. It would be valuable to assess different aspects of behavioral/motor and cognitive response inhibition with multiple tests in specific FND subgroups in future studies.

#### Social cognition

In contrast to our hypotheses, there was no group difference in Bias Point scores on either EBT variant, suggesting that the FMS/FS group did not display enhanced attentional bias toward facial anger or disgust on these tasks. The faster RTs observed on EBT-Anger in the FMS/FS group might reflect hypervigilance to facial anger, as described previously in two studies using emotional Stroop paradigms in FS samples (Bakvis et al., 2009; Pick et al., 2018b). Whilst the previous studies involved subliminal facial stimulus presentations, the CANTAB EBTs present facial stimuli above the threshold of conscious detection. Therefore, the previous tasks invoked preconscious processing whereas the CANTAB EBTs rely on conscious/intentional discrimination between expressions. There may be an implicit, preconscious hypervigilance for angry expressions in FS that is reflected in altered automatic behavioral responses, but that does not influence intentional/voluntary responses.

The lack of impairment on the Emotion Recognition Task in this FMS/FS sample was contrary to our hypotheses and contrasts with a previous report of poorer explicit facial expression recognition in FS (Pick et al., 2016a). Additional studies should explore facial expression processing in more detail in specific FND phenotypes, including further examination of possible preconscious hypervigilance and altered explicit recognition of facial emotions.

### Subjective neurocognitive functioning

#### Task-specific performance ratings

The current FMS/FS sample did not show any overall differences to HCs in their subjective task-specific performance ratings, supporting previous reports of intact local metacognition in FCD (Pennington et al., 2021; Teodoro et al., 2023) and FMS (Bègue et al., 2018; Bhome et al., 2022; Matthews et al., 2020).

One exception was the IEDSS task, on which we observed strong concordance in the HC group between objective outcomes and subjective performance ratings, but only moderate concordance in the FMS/FS group, suggesting possibly reduced accuracy in local metacognition for this task in the latter group. This finding might reflect reduced responsiveness to objective feedback on the IEDSS task, which includes presentation of clear auditory tones to signify correct and erroneous responses on every trial.

The observed correlations between subjective test-specific performance ratings and PHQ-9, B-IPQ and FND symptom scores in the FMS/FS group suggested that self-evaluated underestimation of neurocognitive performance was linked to mood disturbances and illness-related factors, rather than objective performance deficits. It will be valuable to explore local metacognition in more detail in larger FND samples, and to explore further interactions between local metacognitive ratings, FND-related variables, and psychological distress.

#### Daily subjective cognitive functioning

There was a significant elevation in daily cognitive symptoms on the CFQ in the FND sample compared to controls, confirming our hypothesis and strengthening existing evidence (e.g., Heintz et al., 2013; Věchetová et al., 2022). The discordance between objective and subjective neurocognitive functioning in this FMS/FS sample suggests a possible deficit in global metacognition similar to that reported in FCD (Bhome et al., 2022; Teodoro et al., 2023). These results accord with prior studies in which patients with FS underestimated their neurocognitive performance (Breier et al., 1998; Fargo et al., 2004; Prigatano & Kirlin, 2009) and within a broader pattern of findings in other domains in FS/FMS samples, including interoception (Pick et al., 2020), affective reactivity (Pick et al., 2018a) and symptom perception (Kramer et al., 2019; Pareés et al., 2012), in which subjective reports and objective measures diverge (Adewusi et al., 2021).

In this study, daily cognitive complaints (CFQ scores) were associated negatively with task-specific subjective performance ratings for sustained attention and working memory tests in the FMS/FS group, suggesting that global cognitive complaints could be related to inaccurate local metacognitive evaluations for daily tasks involving working memory and attention. Similarly, Bhome et al. (2022) noted an association between local metacognitive bias and global metacognitive scores in FCD.

Here, daily cognitive complaints were also associated with psychological symptoms (depression, dissociation), reminiscent of previous reports in FS/FMS (e.g., Fargo et al., 2004; Věchetová et al., 2022), functional and organic samples (Wagle et al., 1999) and in the healthy population (Larson et al., 1997; Mahoney et al., 1998).

### Strengths and limitations

Strengths of this study were the adoption of a range of objective and subjective neurocognitive measures, and exploration of relationships between neurocognitive outcome variables and clinical features in the FMS/FS group. The automated test battery may have minimized performance-related anxiety that could be heightened when assessments are delivered by a healthcare professional. The FMS/FS and HC groups did not differ significantly in age, sex, handedness, relationship status, ethnicity, education, or intellectual functioning, thereby allowing us to exclude these possible confounds. There were no group differences in sensorimotor or information processing speed and effort that could have unduly influenced our results, also suggesting that medication effects were well-controlled.

Limitations of the study included the lack of clinical controls, inability to obtain trial-level local metacognitive ratings due to the use of an automated test battery, and the use of retrospective self-report scales to assess subjective global cognitive functioning and other background factors (e.g., alexithymia). Furthermore, we administered social cognition tests involving only facial expression processing which limits these findings. Additional affective and social cognition tests could be adopted in subsequent studies (Pick et al., 2019).

The small sample size and resulting limited statistical power may in part explain the lack of group differences observed on the objective neurocognitive tests. However, we presented effect sizes to highlight potentially meaningful effects that did not meet statistical significance. No thorough objective assessment of memory and language functions was included, and further studies are needed to examine the full range of neurocognitive functions in specific FND subgroups.

The mix of primary diagnoses of FMS and FS, alongside other neurological symptoms in this sample prohibited inferences about the neurocognitive profiles associated with specific FND symptoms. The omission of FCD as a specific FND subgroup is also a limitation in this study, given the primary concern of subjective cognitive complaints in that group. Another limitation was the recruitment strategy, which identified participants with FMS/FS via peer-support charities and social media, rather than specialist clinical services. It is possible that some of the additional neurological symptoms reported by the FMS/FS group may have been clinically significant. Future studies might consider including and comparing directly relatively homogenous groups of participants with specific FND phenotypes including FCD, FMS and FS.

## Conclusions

Our preliminary data did not provide evidence of objective deficits in attention and executive functioning, or altered social cognition, in this FMS/FS sample. Nevertheless, this FMS/FS sample reported significant cognitive symptom burden in their daily lives and were less accurate in appraising aspects of their executive functioning. These incongruous findings may be related to psychological symptom burden or metacognitive deficits and resemble similar findings in samples with FCD for whom cognitive symptoms are the primary functional complaint.

These findings are relevant to several mechanistic and neurobiological models of FND which emphasize disrupted attention, executive function and emotion processing. Further research is needed to identify the nature and impact of possible neurocognitive differences in specific FND subgroups and their underlying neurobiological bases. Improved understanding of neurocognitive functioning in FND might accelerate the development of novel interventions for cognitive symptoms in these populations in future.

## Supplementary Material

Supplemental Material

## Data Availability

Data available on reasonable request.

## References

[cit0001] Adewusi, J., Levita, L., Gray, C., & Reuber, M. (2021). Subjective versus objective measures of distress, arousal and symptom burden in patients with functional seizures and other functional neurological symptom disorder presentations: A systematic review. *Epilepsy & Behavior Reports*, 16, 100502. 10.1016/j.ebr.2021.100502 34917921 PMC8669370

[cit0002] American Psychiatric Association. (2013). Diagnostic and statistical manual of mental disorders (5th ed.). 10.1176/appi.books.9780890425596

[cit0003] Bagby, R. M., Parker, J. D., & Taylor, G. J. (1994). The twenty-item Toronto Alexithymia Scale–I. Item selection and cross-validation of the factor structure. *Journal of Psychosomatic Research*, 38(1), 23–32. 10.1016/0022-3999(94)90006-X 8126686

[cit0004] Bakvis, P., Roelofs, K., Kuyk, J., Edelbroek, P. M., Swinkels, W. A., & Spinhoven, P. (2009). Trauma, stress, and preconscious threat processing in patients with psychogenic nonepileptic seizures. *Epilepsia*, 50(5), 1001–1011. 10.1111/j.1528-1167.2008.01862.x 19170739

[cit0005] Ball, H. A., McWhirter, L., Ballard, C., Bhome, R., Blackburn, D. J., Edwards, M. J., Fleming, S. M., Fox, N. C., Howard, R., Huntley, J., Isaacs, J. D., Larner, A. J., Nicholson, T. R., Pennington, C. M., Poole, N., Price, G., Price, J. P., Reuber, M., Ritchie, C., … Carson, A. J. (2020). Functional cognitive disorder: Dementia’s blind spot. *Brain*, 143(10), 2895–2903. 10.1093/brain/awaa224 32791521 PMC7586080

[cit0006] Ball, H. A., Swirski, M., Newson, M., Coulthard, E. J., & Pennington, C. M. (2021). Differentiating functional cognitive disorder from early neurodegeneration: A clinic-based study. *Brain Sciences*, 11(6), 800. 10.3390/brainsci11060800 34204389 PMC8234331

[cit0007] Baron-Cohen, S., Wheelwright, S., Skinner, R., Martin, J., & Clubley, E. (2001). The autism-spectrum quotient (AQ): Evidence from Asperger syndrome/high-functioning autism, males and females, scientists and mathematicians. *Journal of Autism and Developmental Disorders*, 31(1), 5–17. 10.1023/a:1005653411471 11439754

[cit0008] Baslet, G. (2011). Psychogenic non-epileptic seizures: A model of their pathogenic mechanism. *Seizure*, 20(1), 1–13. 10.1016/j.seizure.2010.10.032 21106406

[cit0009] Bègue, I., Blakemore, R., Klug, J., Cojan, Y., Galli, S., Berney, A., Aybek, S., & Vuilleumier, P. (2018). Metacognition of visuomotor decisions in conversion disorder. *Neuropsychologia*, 114, 251–265. 10.1016/j.neuropsychologia.2018.04.018 29698734

[cit0010] Bhome, R., Huntley, J. D., Price, G., & Howard, R. J. (2019). Clinical presentation and neuropsychological profiles of functional cognitive disorder patients with and without co-morbid depression. *Cognitive Neuropsychiatry*, 24(2), 152–164. 10.1080/13546805.2019.1590190 30857470

[cit0011] Bhome, R., McWilliams, A., Price, G., Poole, N. A., Howard, R. J., Fleming, S. M., & Huntley, J. D. (2022). Metacognition in functional cognitive disorder. *Brain Communications*, 4(2), fcac041. 10.1093/braincomms/fcac041 35243345 PMC8889108

[cit0012] Black, L. C., Schefft, B. K., Howe, S. R., Szaflarski, J. P., Yeh, H. S., & Privitera, M. D. (2010). The effect of seizures on working memory and executive functioning performance. *Epilepsy & Behavior: E&B*, 17(3), 412–419. 10.1016/j.yebeh.2010.01.006 20153981

[cit0013] Breier, J. I., Fuchs, K. L., Brookshire, B. L., Wheless, J., Thomas, A. B., Constantinou, J., & Willmore, L. J. (1998). Quality of life perception in patients with intractable epilepsy or pseudoseizures. *Archives of Neurology*, 55(5), 660–665. 10.1001/archneur.55.5.660 9605722

[cit0014] Bridger, R. S., Johnsen, S., & Brasher, K. (2013). Psychometric properties of the Cognitive Failures Questionnaire. *Ergonomics*, 56(10), 1515–1524. 10.1080/00140139.2013.821172 23879800

[cit0015] Briere, J. (2002). *Multiscale Dissociation Inventory professional manual*. Psychological Assessment Resources.

[cit0016] Broadbent, D. E., Cooper, P. F., FitzGerald, P., & Parkes, K. R. (1982). The cognitive failures questionnaire (CFQ) and its correlates. *British Journal of Clinical Psychology*, 21(1), 1–16. 10.1111/j.2044-8260.1982.tb01421.x 7126941

[cit0017] Broadbent, E., Petrie, K. J., Main, J., & Weinman, J. (2006). The brief illness perception questionnaire. *Journal of Psychosomatic Research*, 60(6), 631–637. 10.1016/j.jpsychores.2005.10.020 16731240

[cit0018] Brown, L. B., Nicholson, T. R., Aybek, S., Kanaan, R. A., & David, A. S. (2014). Neuropsychological function and memory suppression in conversion disorder. *Journal of Neuropsychology*, 8(2), 171–185. 10.1111/jnp.12017 23582098

[cit0019] Brown, R. J., & Reuber, M. (2016). Towards an integrative theory of psychogenic non-epileptic seizures (PNES). *Clinical Psychology Review*, 47, 55–70. 10.1016/j.cpr.2016.06.003 27340856

[cit0020] Burmester, B., Leathem, J., & Merrick, P. (2016). Subjective cognitive complaints and objective cognitive function in aging: a systematic review and meta-analysis of recent cross-sectional findings. *Neuropsychology Review*, 26(4), 376–393. 10.1007/s11065-016-9332-2 27714573

[cit0021] Cambridge Cognition. (2019). *CANTAB Connect Research v11. 10*. Cambridge Cognition Limited. https://cambridgecognition.com/digital-cognitive-assessments/

[cit0022] Crumley, J. J., Stetler, C. A., & Horhota, M. (2014). Examining the relationship between subjective and objective memory performance in older adults: A meta-analysis. *Psychology and Aging*, 29(2), 250–263. 10.1037/a0035908 24955993

[cit0023] Demır, S., Çelıkel, F., Taycan, S. E., & Etıkan, İ. (2013). Neuropsychological assessment in conversion disorder. *Turk Psikiyatri Dergisi = Turkish Journal of Psychiatry*, 24(2), 75–83. Konversiyon bozukluğunda nöropsikolojik değerlendirme 23754260

[cit0024] de Paula, J. J., Costa, D. S., Miranda, D. M. D., & Romano-Silva, M. A. (2017). Brazilian version of the Cognitive Failures Questionnaire (CFQ): Cross-cultural adaptation and evidence of validity and reliability. *Brazilian Journal of Psychiatry*, 40(3), 312–315. 10.1590/1516-4446-2017-2227 29236920 PMC6899407

[cit0025] de Vroege, L., Koppenol, I., Kop, W. J., Riem, M. M., & van der Feltz‐cornelis, C. M. (2021). Neurocognitive functioning in patients with conversion disorder/functional neurological disorder. *Journal of Neuropsychology*, 15(1), 69–87. 10.1111/jnp.12206 32223071 PMC8048909

[cit0026] Drane, D. L., Fani, N., Hallett, M., Khalsa, S. S., Perez, D. L., & Roberts, N. A. (2021). A framework for understanding the pathophysiology of functional neurological disorder. *CNS Spectrums*, 26(6), 555–561. 10.1017/S1092852920001789 PMC7930164 32883381

[cit0027] Edwards, M. J., Adams, R. A., Brown, H., Parees, I., & Friston, K. J. (2012). A Bayesian account of ‘hysteria.’ *Brain*, 135(11), 3495–3512. 10.1093/brain/aws129 22641838 PMC3501967

[cit0028] Fargo, J. D., Schefft, B. K., Szaflarski, J. P., Dulay, M. F., Testa, S. M., Privitera, M. D., & Yeh, H.-S. (2004). Accuracy of self-reported neuropsychological functioning in individuals with epileptic or psychogenic nonepileptic seizures. *Epilepsy & Behavior*, 5(2), 143–150. 10.1016/j.yebeh.2003.11.023 15123013

[cit0029] First, M. B., Williams, J. B. W., Karg, R. S., & Spitzer, R. L. (2016). *User’s guide for the SCID-5-CV structured clinical interview for DSM-5® disorders: Clinical version. User’s guide for the SCID-5-CV Structured Clinical Interview for DSM-5® disorders: Clinical version*. American Psychiatric Publishing, Inc.

[cit0030] Forejtová, Z., Serranová, T., Sieger, T., Slovák, M., Nováková, L., Věchetová, G., and Edwards, M. J. (2022). The complex syndrome of functional neurological disorder. *Psychological Medicine*, 1–11. 10.1017/S0033291721005225 34991744

[cit0031] Fray, P. J., Robbins, T. W., & Sahakian, B. J. (1996). Neuorpsychiatyric applications of CANTAB. *International Journal of Geriatric Psychiatry*, 11(4), 329–336. 10.1002/(SICI)1099-1166(199604)11:4<329::AID-GPS453>3.0.CO;2-6

[cit0032] Goldstein, L., Robinson, E., Mellers, J., Stone, J., Carson, A., Chalder, T., Reuber, M., Eastwood, C., Landau, S., McCrone, P., Moore, M., Mosweu, I., Murray, J., Perdue, I., Pilecka, I., Richardson, M. P., & Medford, N. (2021). Psychological and demographic characteristics of 368 patients with dissociative seizures: Data from the CODES cohort. *Psychological Medicine*, 51(14), 2433–2445. 10.1017/S0033291720001051 32389147 PMC8506352

[cit0033] Green, P. (2003). *revised 2005 Medical Symptom Validity Test for Windows: User’s manual and program*.

[cit0034] Green, P., & Flaro, L. (2016). Results from three performance validity tests in children with intellectual disability. *Applied Neuropsychology. Child*, 5(1), 25–34. 10.1080/21622965.2014.935378 25495876

[cit0035] Green, P., Montijo, J., & Brockhaus, R. (2011). High specificity of the word memory test and medical symptom validity test in groups with severe verbal memory impairment. *Applied Neuropsychology*, 18(2), 86–94. 10.1080/09084282.2010.523389 21660760

[cit0036] Hallett, M., Aybek, S., Dworetzky, B. A., McWhirter, L., Staab, J. P., & Stone, J. (2022). Functional neurological disorder: New subtypes and shared mechanisms. *The Lancet Neurology*, 21(6), 537–550. 10.1016/S1474-4422(21)00422-1 35430029 PMC9107510

[cit0037] Hammond-Tooke, G. D., Grajeda, F. T., Macrorie, H., & Franz, E. A. (2018). Response inhibition in patients with functional neurological symptom disorder. *Journal of Clinical Neuroscience*, 56, 38–43. 10.1016/j.jocn.2018.08.005 30145086

[cit0038] Hamouda, K., Senf-Beckenbach, P. A., Gerhardt, C., Irorutola, F., Rose, M., & Hinkelmann, K. (2021). Executive functions and attention in patients with psychogenic nonepileptic seizures compared with healthy controls: A cross-sectional study. *Psychosomatic Medicine*, 83(8), 880–886. 10.1097/PSY.0000000000000981 34292202

[cit0039] Hays, R. D., Sherbourne, C. D., & Mazel, R. M. (1993). The RAND 36-Item Health Survey 1.0. *Health Economics*, 2(3), 217–227. 10.1002/hec.4730020305 8275167

[cit0040] Hedges, L. V., & Olkin, I. (1985). *Statistical methods for meta-analysis*. Academic Press.

[cit0041] Heintz, C. E., van Tricht, M. J., van der Salm, S. M., van Rootselaar, A. F., Cath, D., Schmand, B., & Tijssen, M. A. (2013). Neuropsychological profile of psychogenic jerky movement disorders: Importance of evaluating non-credible cognitive performance and psychopathology. *Journal of Neurology, Neurosurgery & Psychiatry*, 84(8), 862–867. 10.1136/jnnp-2012-304397 23418216

[cit0042] Howell, D. C. (1997). *Statistical methods for psychology* (4th) edn. Duxbury.

[cit0043] Howe, L., & Loring, D. (2008). Classification accuracy and predictive ability of the medical symptom validity test’s dementia profile and general memory impairment profile. *The Clinical Neuropsychologist*, 28, 1–14. 10.1080/13854040801945060 18609326

[cit0044] IBM. (2021). *IBM SPSS statistics for windows (Version 28.0)*.

[cit0045] Jungilligens, J., Paredes-Echeverri, S., Popkirov, S., Barrett, L. F., & Perez, D. L. (2022). A new science of emotion: Implications for functional neurological disorder. *Brain*, 145(8), 2648–2663. 10.1093/brain/awac204 35653495 PMC9905015

[cit0046] Jungilligens, J., Wellmer, J., Schlegel, U., Kessler, H., Axmacher, N., & Popkirov, S. (2020). Impaired emotional and behavioural awareness and control in patients with dissociative seizures. *Psychological Medicine*, 50(16), 2731–2739. 10.1017/s0033291719002861 31625504

[cit0047] Keynejad, R. C., Fenby, E., Pick, S., Moss-Morris, R., Hirsch, C., Chalder, T., Hughes, A. M., & Nicholson, T. R. (2020). Attentional processing and interpretative bias in functional neurological disorder. *Psychosomatic Medicine*, 82(6), 586–592. 10.1097/PSY.0000000000000821 32541544

[cit0048] Kozlowska, K., Palmer, D. M., Brown, K. J., Scher, S., Chudleigh, C., Davies, F., & Williams, L. M. (2015). Conversion disorder in children and adolescents: A disorder of cognitive control. *Journal of Neuropsychology*, 9(1), 87–108. 10.1111/jnp.12037 24405496

[cit0049] Kramer, G., Dominguez-Vega, Z. T., Laarhoven, H. S., Brandsma, R., Smit, M., van der Stouwe, A. M., Elting, J. W. J., Maurits, N. M., Rosmalen, J. G., & Tijssen, M. A. (2019). Similar association between objective and subjective symptoms in functional and organic tremor. *Parkinsonism & Related Disorders*, 64, 2–7. 10.1016/j.parkreldis.2019.05.026 31151787

[cit0050] Kroenke, K., & Spitzer, R. L. (2002). The PHQ-9: A new depression diagnostic and severity measure. *Psychiatric Annals*, 32(9), 509–515. 10.3928/0048-5713-20020901-06

[cit0051] Kroenke, K., Spitzer, R. L., & Williams, J. B. (2001). The PHQ-9: Validity of a brief depression severity measure. *Journal of General Internal Medicine*, 16(9), 606–613. 10.1046/j.1525-1497.2001.016009606.x 11556941 PMC1495268

[cit0052] Larson, G. E., Alderton, D. L., Neideffer, M., & Underhill, E. (1997). Further evidence on dimensionality and correlates of the Cognitive Failures Questionnaire. *British Journal of Psychology*, 88(1), 29–38. 10.1111/j.2044-8295.1997.tb02618.x

[cit0053] Mahoney, A. M., Dalby, J. T., & King, M. C. (1998). Cognitive failures and stress. *Psychological Reports*, 82(3_suppl), 1432–1434. 10.2466/pr0.1998.82.3c.1432 9709545

[cit0054] Matthews, J., Nagao, K., Ding, C., Newby, R., Kempster, P., & Hohwy, J. (2020). Raised visual contrast thresholds with intact attention and metacognition in functional motor disorder. *Cortex; a Journal Devoted to the Study of the Nervous System and Behavior*, 125, 161–174. 10.1016/j.cortex.2019.12.009 31991241

[cit0055] McWhirter, L., King, L., McClure, E., Ritchie, C., Stone, J., & Carson, A. (2022). The frequency and framing of cognitive lapses in healthy adults. *CNS Spectrums*, 27(3), 331–338. 10.1017/S1092852920002096 33478616

[cit0056] McWhirter, L., Ritchie, C., Stone, J., & Carson, A. (2022). Identifying functional cognitive disorder: A proposed diagnostic risk model. *CNS Spectrums*, 27(6), 754–763. 10.1017/S1092852921000845 34533113

[cit0057] Mundt, J. C., Marks, I. M., Shear, M. K., & Greist, J. H. (2002). The work and social adjustment scale: A simple measure of impairment in functioning. *British Journal of Psychiatry*, 180(5), 461–464. 10.1192/bjp.180.5.461 11983645

[cit0058] Myers, L., Zeng, R., Perrine, K., Lancman, M., & Lancman, M. (2014). Cognitive differences between patients who have psychogenic nonepileptic seizures (PNESs) and posttraumatic stress disorder (PTSD) and patients who have PNESs without PTSD. *Epilepsy & Behavior*, 37, 82–86. 10.1016/j.yebeh.2014.06.009 25010320

[cit0059] Nijenhuis, E. R., Spinhoven, P., Van Dyck, R., Van Der Hart, O., & Vanderlinden, J. (1996). The development and psychometric characteristics of the Somatoform Dissociation Questionnaire (SDQ-20). *Journal of Nervous and Mental Disease*, 184(11), 688–694. 10.1097/00005053-199611000-00006 8955682

[cit0060] Nijenhuis, E. R., Van der Hart, O., & Kruger, K. (2002). The psychometric characteristics of the Traumatic Experiences Checklist (TEC): First findings among psychiatric outpatients. *Clinical Psychology & Psychotherapy: An International Journal of Theory & Practice*, 9(3), 200–210. 10.1002/cpp.332

[cit0061] O’Brien, F. M., Fortune, G. M., Dicker, P., O’Hanlon, E., Cassidy, E., Delanty, N., Garavan, H., & Murphy, K. C. (2015). Psychiatric and neuropsychological profiles of people with psychogenic nonepileptic seizures. *Epilepsy & Behavior*, 43, 39–45. 10.1016/j.yebeh.2014.11.012 25553390

[cit0062] Özer Çelik, A., Kurt, P., Yener, G., Alkin, T., Öztura, İ., & Baklan, B. (2015). Comparison of cognitive impairment between patients having epilepsy and psychogenic nonepileptic seizures. *Noro Psikiyatri Arsivi*, 52(2), 163–168. 10.5152/npa.2015.7290 28360698 PMC5353192

[cit0063] Pareés, I., Saifee, T. A., Kassavetis, P., Kojovic, M., Rubio-Agusti, I., Rothwell, J. C., Bhatia, K. P., & Edwards, M. J. (2012). Believing is perceiving: Mismatch between self-report and actigraphy in psychogenic tremor. *Brain*, 135(Pt 1), 117–123. 10.1093/brain/awr292 22075068

[cit0064] Pennington, C., Ball, H., Swirski, M., Newson, M., & Coulthard, E. (2021). Metacognitive performance on memory and visuospatial tasks in functional cognitive disorder. *Brain Sciences*, 11(10), 1368. 10.3390/brainsci11101368 34679432 PMC8533868

[cit0065] Pennington, C., Hayre, A., Newson, M., Coulthard, E., Tales, A., Jessen, F., Butler, C., Wilcock, G., Phillips, J., & Bayer, T. (2015). Functional cognitive disorder: A common cause of subjective cognitive symptoms. *Journal of Alzheimer’s Disease: JAD*, 48(Suppl 1), S19–S24. 10.3233/JAD-150182 26402086

[cit0066] Pennington, C., Newson, M., Hayre, A., & Coulthard, E. (2015). Functional cognitive disorder: What is it and what to do about it? *Practical Neurology*, 15(6), 436–444. 10.1136/practneurol-2015-001127 26271265

[cit0067] Perez, D. L., Barsky, A. J., Daffner, K., & Silbersweig, D. A. (2012). Motor and somatosensory conversion disorder: A functional unawareness syndrome? *The Journal of Neuropsychiatry and Clinical Neurosciences*, 24(2), 141–151. 10.1176/appi.neuropsych.11050110 22772662

[cit0068] Pick, S., Goldstein, L. H., Perez, D. L., & Nicholson, T. R. (2019). Emotional processing in functional neurological disorder: A review, biopsychosocial model and research agenda. *Journal of Neurology, Neurosurgery & Psychiatry*, 90(6), 704–711. 10.1136/jnnp-2018-319201 30455406 PMC6525039

[cit0069] Pick, S., Mellers, J. D., & Goldstein, L. H. (2016a). Explicit facial emotion processing in patients with dissociative seizures. *Psychosomatic Medicine*, 78(7), 874–885. 10.1097/psy.0000000000000327 27187848

[cit0070] Pick, S., Mellers, J. D., & Goldstein, L. H. (2018a). Autonomic and subjective responsivity to emotional images in people with dissociative seizures. *Journal of Neuropsychology*, 12(2), 341–355. 10.1111/jnp.12144 29285879 PMC6001553

[cit0071] Pick, S., Mellers, J. D., & Goldstein, L. H. (2018b). Implicit attentional bias for facial emotion in dissociative seizures: Additional evidence. *Epilepsy & Behavior*, 80, 296–302. 10.1016/j.yebeh.2018.01.004 29402630

[cit0072] Pick, S., Rojas-Aguiluz, M., Butler, M., Mulrenan, H., Nicholson, T. R., & Goldstein, L. H. (2020). Dissociation and interoception in functional neurological disorder. *Cognitive Neuropsychiatry*, 25(4), 294–311. 10.1080/13546805.2020.1791061 32635804

[cit0073] Prigatano, G. P., & Kirlin, K. A. (2009). Self-appraisal and objective assessment of cognitive and affective functioning in persons with epileptic and nonepileptic seizures. *Epilepsy & Behavior*, 14(2), 387–392. 10.1016/j.yebeh.2008.12.001 19126438

[cit0074] R Core Team. (2021). *R: A language and environment for statistical computing*. https://www.R-project.org/

[cit0075] Robbins, T. (1994). Cambridge neuropsychological test automated battery (CANTAB): Utility and validation. IEE colloquium on computer-aided tests of drug effectiveness.

[cit0076] Robbins, T. W., James, M., Owen, A. M., Sahakian, B. J., Lawrence, A. D., McInnes, L., & Rabbitt, P. M. (1998). A study of performance on tests from the CANTAB battery sensitive to frontal lobe dysfunction in a large sample of normal volunteers: Implications for theories of executive functioning and cognitive aging. *Journal of the International Neuropsychological Society*, 4(5), 474–490. 10.1017/S1355617798455073 9745237

[cit0077] Roelofs, K., van Galen, G. P., Eling, P., Keijsers, G. P. J., & Hoogduin, C. A. L. (2003). Endogenous and exogenous attention in patients with conversion paresis. *Cognitive Neuropsychology*, 20(8), 733–745. 10.1080/02643290342000069 20957591

[cit0078] Schönenberg, M., Jusyte, A., Höhnle, N., Mayer, S. V., Weber, Y., Hautzinger, M., & Schell, C. (2015). Theory of mind abilities in patients with psychogenic nonepileptic seizures. *Epilepsy & Behavior*, 53, 20–24. 10.1016/j.yebeh.2015.09.036 26515154

[cit0079] Simani, L., Roozbeh, M., Rostami, M., Pakdaman, H., Ramezani, M., & Asadollahi, M. (2020). Attention and inhibitory control deficits in patients with genetic generalized epilepsy and psychogenic nonepileptic seizure. *Epilepsy & Behavior*, 102, 106672. 10.1016/j.yebeh.2019.106672 31739099

[cit0080] Spitzer, R. L., Kroenke, K., Williams, J. B., & Löwe, B. (2006). A brief measure for assessing generalized anxiety disorder: The GAD-7. *Archives of Internal Medicine*, 166(10), 1092–1097. 10.1001/archinte.166.10.1092 16717171

[cit0081] Stone, J., Pal, S., Blackburn, D., Reuber, M., Thekkumpurath, P., Carson, A., Tales, A., Jessen, F., Butler, C., Wilcock, G., Phillips, J., & Bayer, T. (2015). Functional (psychogenic) cognitive disorders: A perspective from the neurology clinic. *Journal of Alzheimer’s Disease*, 48(s1), S5–S17. 10.3233/JAD-150430 26445274

[cit0082] Strutt, A. M., Hill, S. W., Scott, B. M., Uber-Zak, L., & Fogel, T. G. (2011). A comprehensive neuropsychological profile of women with psychogenic nonepileptic seizures. *Epilepsy & Behavior*, 20(1), 24–28. 10.1016/j.yebeh.2010.10.004 21075059

[cit0083] Teodoro, T., Edwards, M. J., & Isaacs, J. D. (2018). A unifying theory for cognitive abnormalities in functional neurological disorders, fibromyalgia and chronic fatigue syndrome: Systematic review. *Journal of Neurology, Neurosurgery, and Psychiatry*, 89(12), 1308–1319. 10.1136/jnnp-2017-317823 29735513 PMC6288708

[cit0084] Teodoro, T., Koreki, A., Chen, J., Coebergh, J., Poole, N., Ferreira, J. J., Edwards, M. J., & Isaacs, J. D. (2023). Functional cognitive disorder affects reaction time, subjective mental effort and global metacognition. *Brain: a Journal of Neurology*, 146(4), 1615–1623. 10.1093/brain/awac363 36200349

[cit0085] Tyson, B. T., Baker, S., Greenacre, M., Kent, K. J., Lichtenstein, J. D., Sabelli, A., & Erdodi, L. A. (2018). Differentiating epilepsy from psychogenic nonepileptic seizures using neuropsychological test data. *Epilepsy & Behavior*, 87, 39–45. 10.1016/j.yebeh.2018.08.010 30172082

[cit0086] Věchetová, G., Nikolai, T., Slovák, M., Forejtová, Z., Vranka, M., Straková, E., Teodoro, T., Růžička, E., Edwards, M. J., & Serranová, T. (2022). Attention impairment in motor functional neurological disorders: A neuropsychological study. *Journal of Neurology*, 269(11), 5981–5990. 10.1007/s00415-022-11211-x 35842882

[cit0087] Věchetová, G., Slovák, M., Kemlink, D., Hanzlíková, Z., Dušek, P., Nikolai, T., Růžička, E., Edwards, M. J., & Serranová, T. (2018). The impact of non-motor symptoms on the health-related quality of life in patients with functional movement disorders. *Journal of psychosomatic research*, 115, 32–37. 10.1016/j.jpsychores.2018.10.001 30470314

[cit0088] Verrel, J., Chwolka, F., Filevich, E., Moyé, J., Paulus, T., Zittel, S., Bäumer, T., Münchau, A., & Weissbach, A. (2023). Impaired metacognition of voluntary movement in functional movement disorder. *Movement Disorders: Official Journal of the Movement Disorder Society*, 38(3), 435–443. 10.1002/mds.29303 36606550

[cit0089] Voon, V., Ekanayake, V., Wiggs, E., Kranick, S., Ameli, R., Harrison, N. A., & Hallett, M. (2013). Response inhibition in motor conversion disorder. *Movement Disorders*, 28(5), 612–618. 10.1002/mds.25435 23554084 PMC4096145

[cit0090] Wagle, A. C., Berrios, G. E., & Ho, L. (1999). The cognitive failures questionnaire in psychiatry. *Comprehensive Psychiatry*, 40(6), 478–484. 10.1016/s0010-440x(99)90093-7 10579381

[cit0091] Wallace, J. C., Kass, S. J., & Stanny, C. J. (2002). The cognitive failures questionnaire revisited: Dimensions and correlates. *The Journal of General Psychology*, 129(3), 238–256. 10.1080/00221300209602098 12224809

[cit0092] Wechsler, D. (2011). *Wechsler Abbreviated Scale of Intelligence -* *(Second Edition)* ed.). NCS Pearson, Inc.

