# Supplementary material for: Objective and subjective neurocognitive functioning in functional motor symptoms and functional seizures: preliminary findings
Source: J Clin Exp Neuropsychol. 2023 Sep 19;45(10):970–87. doi: 10.1080/13803395.2023.2245110 (PMC11057846; doi:10.1080/13803395.2023.2245110)
Supplement: Supplemental Material [file NCEN_A_2245110_SM7532.docx]

**Supplementary Materials**

**Supplementary Table 1. CANTAB Connect outcome variables**

| **Task** | **Outcome variables** | **Description** | **Interpretation** |
| --- | --- | --- | --- |
| **Motor Screening Test** | Motor Mean Latency | The mean latency for a participant to correctly respond to the stimulus on screen during assessed trials, in milliseconds. | Higher scores indicate slower responding (poorer performance). |
| **Reaction Time** | Reaction Time | The median duration to release the response button after stimulus. | Higher scores indicate slower responding (poorer performance). |
|  | Movement Time | Median time to release the response button and select the target stimulus. | Higher scores indicate slower sensorimotor speed (poorer performance). |
|  | Total Error Score | The total number of trials where the participant made any form of response error, including inaccurate responses, incorrect location, omission and premature errors, as well as use of multiple fingers and dragging a finger outside of a response box. Calculated across all assessment trials. | Higher scores indicate more errors (poorer performance). |
| **Rapid Visual Information Processing** | Response Latency | The median response latency for correct responses, in milliseconds. | Higher scores indicate longer response latency (poorer performance). |
|  | RVP Ability | A signal detection measure of the participant’s overall ability in detecting target sequences. | Expected range = 0.00 to 1.00 (bad to good) |
|  | Probability of False Alarm | The number of sequence presentations that were false alarms divided by the number of sequence presentations that were false alarms plus the number of sequence presentations that were correct rejections. | Higher scores indicate greater likelihood of false alarms (poorer performance). |
|  | Probability of Hit | The number of target sequences that were correctly responded to, divided by the number of target sequences. | Higher scores indicate greater probability of hits (superior performance). |
| **Spatial Span** | Forward Span Length | The longest sequence successfully recalled by the participant. | Higher scores indicate longer spatial span length (superior performance). |
|  | Reverse Span Length |  |  |
| **Intra-Extra Dimensional Set Shift** | Total Errors | The total number of trials for which an incorrect response was made within the response window, across all assessed trials. | Higher scores indicate more errors (poorer performance). |
|  | Adjusted Errors | The total number of times that the participant chose a wrong stimulus, adjusted for stages that were not reached.  Total Errors Adjusted = Total Errors (problems reached) + [(number of unreached problems) * (25)]. | Higher scores indicate more (adjusted) errors (poorer performance). |
|  | Trials completed | The number of trials completed on all attempted stages. | Higher scores indicate more trials completed (poorer performance) |
|  | Adjusted trials completed | The number of trials completed on all attempted stages with an adjustment for stages not reached.    Total Trials Adjusted = Total Trials (problems reached) + [(number of unreached problems) * (50)] | Higher scores indicate more trials completed (poorer performance) |
|  | Completed Stage Errors | The total number of errors made on stages that were completed. | Higher scores indicate more errors (poorer performance). |
|  | Stages Completed | The total number of stages that were completed successfully (0-9) | Higher scores indicate more stages completed (superior performance). |
|  | Response Latency | The sum of a participant’s response times (milliseconds) across all trials. | Higher scores indicate slower responses (poorer performance). |
| **Stop Signal Task** | Stop Signal Reaction Time | An estimate of time where an individual can successfully inhibit their responses 50% of the time. Calculated from the length of time between the ‘Go’ and ‘Stop’ stimuli at which the participant can successfully inhibit their response on 50% of trials. | Higher scores indicate slower response inhibition (poorer performance). |
|  | Errors: Go Trials | The total number of trials where the participant responded incorrectly to the direction of the arrow stimulus on a Go trial. | Higher scores indicate more errors (poorer performance). |
|  | Errors: Stop Trials | The total number of trials where the subject responded incorrectly to the direction of the arrow stimulus on a Stop trial. | Higher scores indicate more errors (poorer performance). |
|  | Missed Trials | The total number of trials which were missed by the subject. | Higher scores indicate more missed trials (poorer performance). |
| **Emotional Bias Task** | Bias Point | The proportion of assessed trials where the subject selected 'Happy', adjusted to a scale of 0 to 15.  (Number of assessed trials selected as 'Happy'/Number of all assessed trials) x 15 | Higher scores indicate a bias towards choosing 'Happy'. |
|  | Reaction Time | Mean / median total reaction time.  Reaction time for happy selections. | Higher scores indicate slower responding (poorer performance). |
| **Emotional Recognition Task** | Total reaction time | The median latency for a participant to select an emotion after being presented with a stimulus, across all trials. | Higher scores indicate slower responding (poorer performance). |
|  | Reaction time by emotion | The median latency for a participant to select an emotion after being presented with a stimulus for each emotion category. | Higher scores indicate slower responding (poorer performance). |
|  | Total Hits | The total number of correct responses (emotion selection) the participant made, across all trials. | Higher scores indicate more hits (superior performance). |
|  | Hits by emotion | The total number of correct responses (emotion selection) the participant made for each emotion category. | Higher scores indicate more hits (superior performance). |
|  | Unbiased Hit Rate | The unbiased hit rate ensures that recognition accuracy of the emotional categories is not influenced by response guessing or response bias effects. | Higher scores indicate more hits (superior performance). |
|  | False alarms | The number of times a participant erroneously selects an emotion label across all trials. | Higher scores indicate more false alarms (poorer performance). |

**Supplementary Table 2. Clinical self-report measures: Group comparison statistics**

| **Questionnaire** | **Description** | **Scores by group** | | **Comparison statistics** |
| --- | --- | --- | --- | --- |
|  |  | **FND (Total n=16)** | **HCs (Total n=17)** |  |
| **Functional Neurological Symptoms Questionnaire** | A bespoke questionnaire assessing the presence, frequency, severity and impact of FND symptoms over the preceding week. The measure yields scores for: total number of FND symptoms, average severity (1-7) and average impact (1-7) – higher scores indicate more symptoms and greater severity/impact. The nature of the symptoms is also recorded (e.g., seizure, tremor, dizziness, etc). | See Table 2 | - | - |
| **Patient Health Questionnaire – 15 (Kroenke et al., 2002):**  **M (SD)** | Fifteen items assess the frequency of common somatic symptoms over the previous four weeks. Scores range from 0-30 – higher scores indicate more somatic symptoms. | 13.5 (4.2) | 3.2 (2.4) | t(23.9)=-8.6, p<0.001, g=2.97 |
| **Patient Health Questionnaire – 9 (Kroenke et al., 2001):**  **Mdn (IQR)** | Nine items measure the frequency of depressive symptoms over the past two weeks. Scores range from 0-27 – higher scores indicate elevated depressive symptoms. | 12.0 (8.75) | 1.0 (3.0) | W=16.5, p<0.001, r=0.75 |
| **Generalised Anxiety Disorder – 7 (Spitzer et al., 1999):**  **Mdn (IQR)** | Seven items assess the frequency of generalised anxiety symptoms in the past two weeks. Scores range from 0-21 – higher scores indicate more anxiety. | 8.5 (8.5) | 2.0 (4.0) | W=42.0, p<0.001, r=0.59 |
| **Multiscale Dissociation Inventory (Briere, 2002): Mdn (IQR)** | A 30-item measure of the frequency of several forms of psychological dissociation over the preceding month. Raw scores are converted to T-scores (presented here). T-scores range from 0-170 – higher scores indicate greater dissociative symptomology. | **DENG**=74.0 (49.0)  **DEPR**=60.5 (81.2)  **DERL**=57.0 (46.8)  **ECON**=46.0 (10.5)  **MEMD**=64.5 (35.8)  **IDDIS**=47.0 (0.0) | **DENG**=52.0 (8.0)  **DEPR**=47 (0.0)  **DERL**=46.0 (0.0)  **ECON**=46.0 (4.0)  **MEMD**=52.0 (7.0)  **IDDIS**=47.0 (0.0) | ***DENG**: W=65.0, p=0.01, r=0.45  ***DEPR**: U=59.5, p<0.001, r=0.612  ***DERL:** U=66.5, p=0.003, r=0.52  **ECON:** W=130.5, p=0.81, r=0.04  ***MEMD:** W=67.0, p=0.01, r=0.45  **IDDIS:** U=110.5, p=0.07, r=0.32 |
| **Somatoform Dissociation Questionnaire – 20 (Nijenhuis et al., 1996):**  **Mdn (IQR)** | Twenty items examining the extent of various somatoform symptoms in the last year (e.g., sensory disturbances, speech/swallowing difficulties, pain). Scores range from 20-100 – higher scores indicate greater somatoform dissociation. | 30.0 (9.25) | 20.0 (0.0) | W=15, p<.001, r=0.80 |
| **Toronto Alexithymia Scale – 20 (Bagby et al., 1994):**  **M (SD)** | A 20-item measure of difficulties in emotional processing (i.e., identification/description of emotions, external cognitive orientation). Scores range from 20-100 – higher scores indicate greater alexithymia. | 53.3 (10.5) | 42.0 (9.9) | t(31)=-3.17, p=.002, g=1.08 |
| **Autistic Spectrum Quotient (Baron-Cohen et al., 2001):**  **Mdn (IQR)** | A 50-item scale assessing the presence of autistic spectrum traits (e.g., social communication difficulties, repetitive behaviours). Scores range from 0-50 on five subscales – higher scores indicate increased autistic spectrum traits. | 20.5 (10.5) | 16 (8) | W=100, p=.20, r=0.23 |
| **Traumatic Experiences Checklist (Nijenhuis et al., 2002):**  **Mdn (IQR)** | A 33-item measure of lifetime traumatic experiences and their impact (e.g., bullying, life threatening illness, childhood abuse and neglect). Due to ethical concerns, we used a 29-item version, omitting the final four items which probe further details of abuse/maltreatment disclosures. Total scores ranged from 0-29 and impact scores for individual events ranged from 1-5. Higher scores signify greater trauma burden and impact. | **Total =**4.0 (5.25)  **Impact=**13.0 (15.5) | **Total=**2.0 (3.0)  **Impact=**8.0 (11.0) | **Total:** W=83, p=0.05, r=0.34  **Impact:** W=83, p=0.06, r=0.33 |
| **Short Form Health Survey – 36 (Hays et al., 1993)** | A 36-item scale capturing data on health-related quality of life across eight domains. Subscale scores range from 0-100 - higher scores indicate better quality of life. | **PF:** Mdn=40 (IQR=22.5)  **RLP:** Mdn=0.0 (IQR=0.0)  **MH:** Mdn=58.0 (IQR=25.0)  **RLE:** Mdn=16.7 (IQR=100.0)  **E/V:** M=25.9 (SD=18.0)  **Pain:** Mdn=33.8 (IQR=25.6)  **GH:** M=34.1 (SD=19.6)  **SF:** Mdn=37.5 (IQR=37.5) | **PF:** Mdn=95.0 (IQR=5.0)  **RLP:** Mdn= 100.0 (IQR=0.0)  **MH:** Mdn=84.0 (IQR=16.0)  **RLE:** Mdn=100.0 (IQR=33.3)  **E/V:** M=65.3 (SD=13.3)  **Pain:** Mdn=100.0 (IQR=22.5)  **GH:** M=74.1 (SD=14.5)  **SF:** Mdn=100.0 (IQR=25.0) | ***PF:** W=272, p<0.001, r=0.86  ***RLP:** W=259.5, p<0.001, r=0.84  ***MH:** W=223.5, p=0.002, r=0.55  ***RLE:** W=197, p=0.02, r=0.42  ***E/V:** t(31)=7.18, p<0.001, g=2.44  ***Pain:** W=257.5, p<0.001, r=0.77  ***GH:** t(31)=6.70, p<0.001, g=2.28  ***SF:** W=248.5, p<0.001, r=0.72 |
| **Work & Social Adjustment Scale (Mundt et al., 2002):**  **Mdn (IQR)** | A brief 5-item measure of general functioning in occupational and social domains. Scores range from 0-40 – higher scores denote worse functioning. | 25.5 (8.5) | 1.0 (4.5)  (n=14) | W=1.0, p<0.001, r=0.85 |
| **Brief Illness Perception Questionnaire (Broadbent et al., 2006):**  **M (SD)** | Nine items assessing illness-related cognitions (e.g., perceived chronicity, causes, expectations about treatment efficacy). Each item is scored from 0-10 – higher scores represent more threatening illness representations. A ninth item requires respondents to indicate three perceived causes of their illness in rank order. | **Coherence:** 4.56 (2.83)  **Consequences:** 7.38 (2.13)  **Identity:** 7.19 (2.10)  **Illness Concern:** 6.47 (1.96)  **Personal Control:** 6.6 (1.92)  **Timeline:** 7.12 (3.03)  **Treatment Control:** 6.13 (2.17)  **Emotional Response:** 6.88 (2.75)  **Illness threat:** 52.4 (12.6) | - | - |

Key: DEN|G=disengagement; DEPR=depersonalisation; DERL=derealisation; E/V=energy/vitality; ECON=emotional constriction; GH=general health; IDDIS=identity dissociation; IQR=interquartile range; M=mean; Mdn=median; MEMD=memory disturbance; MH=mental health; PF=physical functioning; RLE=emotional role limitations; RLP=physical role limitations; SD=standard deviation; SF=social functioning

*****Significant after Holm-Bonferroni correction

**Supplementary Table 3. Functional Neurological Symptoms Questionnaire**

Please look at the symptoms in the table below and tell us whether you have experienced these functional neurological symptoms in the **past week.** If you mark yes to indicate that the symptom was present in the past week, please complete the additional columns to tell us **how frequent** the symptoms were, **how severe** (intense) they were, and **how much impact** they had on you.

When rating the average **severity** of symptoms, please choose a number from 1 to 7, where **1=Symptom not present; 2=Minimal; 3=Mild; 4=Moderate; 5=Moderately severe; 6=Severe; 7=Very severe**.

When rating the **impact** of symptoms, please choose a number from 1 to 7, where **1=No impact at all; 2=Minimal impact; 3=Mild impact; 4=Moderate impact; 5=Moderately severe impact; 6=Severe impact; 7=Very severe impact**.

| **FND Symptom** | **Present?**  (circle or bold) | **Frequency**  (circle or bold) | **Average severity**  **(1-7)** | **Average impact**  **(1-7)** |
| --- | --- | --- | --- | --- |
| Weakness | Yes / No | Constant / daily / weekly / less than weekly |  |  |
| Tremor | Yes / No | Constant / daily / weekly / less than weekly |  |  |
| Dystonia (muscle spasms / fixed postures) | Yes / No | Constant (1) / daily (2) / weekly (3) / less than weekly (4) |  |  |
| Walking / mobility difficulties | Yes / No | Constant / daily / weekly / less than weekly |  |  |
| Myoclonus (muscle jerks) | Yes / No | Constant / daily / weekly / less than weekly |  |  |
| Seizures* | Yes / No | Number of seizures in the last week: |  |  |
| Numbness (loss of feeling) | Yes / No | Constant / daily / weekly / less than weekly |  |  |
| Visual disturbances | Yes / No | Constant / daily / weekly / less than weekly |  |  |
| Sensitivity to light/sound | Yes / No | Constant / daily / weekly / less than weekly |  |  |
| Dizziness | Yes / No | Constant / daily / weekly / less than weekly |  |  |
| Speech / swallowing difficulties | Yes / No | Constant / daily / weekly / less than weekly |  |  |
| Cognitive difficulties (e.g., brain fog, memory lapses) | Yes / No | Constant / daily / weekly / less than weekly |  |  |
| Other FND symptoms | Details: | Constant / daily / weekly / less than weekly |  |  |

Please tell us which FND symptom(s) is most severe and has the most impact on you:

…............................................................................................................................................

*If you experience FND seizures, do you have warning symptoms? Yes / No

*If you experience warning symptoms before an FND seizure, what is the earliest or most consistent symptom(s) that you experience?

…..................................................................................................................................................................................................................................................................................................

**Supplementary Table 4. Mental and physical health diagnoses**

**and medication**

|  | **FND**  **(n = 16)** | **Control**  **(n = 17)** |
| --- | --- | --- |
|  | ***n (%)*** | ***n (%)*** |
| **Current mental health diagnoses** | 10 (63) | 1 (6) |
| ***Anxiety*** | 7 (44) |  |
| ***Borderline Personality Disorder*** | 1 (6) |  |
| ***Depression*** | 8 (50) |  |
| ***Panic attacks*** | 2 (13) |  |
| ***Post-natal depression*** |  | 1 (6) |
| ***Post-Traumatic Stress Disorder*** | 1 (6) |  |
| ***Seasonal Affective Disorder*** |  | 1 (6) |
| **Current physical health diagnoses** | 11 (69) | 4 (24) |
| ***Allergies*** | 1 (6) |  |
| ***Asthma*** | 2 (13) | 1 (6) |
| ***Chronic pain*** | 1 (6) |  |
| ***Eczema*** |  | 1 (6) |
| ***Ehlers-Danlos Syndrome*** | 1 (6) |  |
| ***Endometriosis*** | 1 (6) |  |
| ***Fibromyalgia*** | 2 (13) |  |
| ***Frequent fainting*** | 1 (6) |  |
| ***Hyperthyroidism*** |  | 1 (6) |
| ***Hemicrania continua*** | 1 (6) |  |
| ***Herpes*** | 1 (6) |  |
| ***Hepatitis B*** | 1 (6) |  |
| ***Irritable Bowel Syndrome*** | 2 (13) | 1 (6) |
| ***Migraines*** | 1 (6) |  |
| ***Sciatica*** |  | 1 (6) |
| ***Vasovagal syncope*** | 1 (6) |  |
| **Medication (% yes)** | 15 (94) | 5 (29) |
| ***Antidepressant*** | 9 (56) | 1 (6) |
| ***Antihistamine*** | 1 (6) | 2 (12) |
| ***Antipsychotic*** | 1 (6) |  |
| ***Anti-convulsant*** | 6 (38) |  |
| ***Anti-migraine*** | 1 (6) |  |
| ***Anti-asthmatic*** | 2 (13) |  |
| ***Anxiolytic*** | 4 (25) |  |
| ***Anti-spasmodic*** |  | 1 (6) |
| ***Anti-thyroid agent*** |  | 1 (6) |
| ***Anti-constipation*** | 2 (13) |  |
| ***Botox*** | 1 (6) |  |
| ***CBD*** | 2 (13) |  |
| ***Contraceptive*** | 1 (6) | 1 (6) |
| ***Dopamine agonist*** | 1 (6) |  |
| ***HRT*** | 1 (6) |  |
| ***High blood pressure*** | 1 (6) |  |
| ***Lipophilic/hydrophilic*** | 1 (6) |  |
| ***Levothyroxine*** | 1 (6) |  |
| ***Muscle relaxant*** | 1 (6) |  |
| ***NRTI*** | 1 (6) |  |
| ***NSAID*** | 2 (13) |  |
| ***Opiate analgesic*** | 4 (25) |  |
| ***Paracetamol*** | 1 (6) |  |
| ***PPI*** | 2 (13) |  |
| ***Selenium*** |  | 1 (6) |

*Key:* FND=functional neurological disorder; HRT=hormone replacement therapy;

NRTI=nucleotide reverse transcriptase inhibitors; CBD=cannabidiol;

NSAID=non-steroidal anti-inflammatory drug; PPI=proton pump inhibitor
